# Methionine-35 of A**β**(1–42): Importance for Oxidative Stress in Alzheimer Disease

**DOI:** 10.4061/2011/198430

**Published:** 2011-06-04

**Authors:** D. Allan Butterfield, Rukhsana Sultana

**Affiliations:** ^1^Department of Chemistry, University of Kentucky, Lexington, KY 40506-0055, USA; ^2^Center of Membrane Sciences, University of Kentucky, Lexington, KY 40506-0055, USA; ^3^Sanders-Brown Center on Aging, University of Kentucky, Lexington, KY 40506-0055, USA

## Abstract

Alzheimer disease (AD) is an age-related progressive neurodegenerative disorder. This devastating disease is characterized by the presence of senile plaques (SP), neurofibrillary tangles (NFTs), and loss of synapses. Amyloid beta-peptide 1–42 (A**β**(1–42)) is the main component of SP and is pivotal to AD pathogenesis. Brain of subjects with AD and arguably its earliest manifestation, mild cognitive impairment (MCI), demonstrate increased levels of oxidative stress markers. Our laboratory combined these two aspects of AD and MCI and proposed the A**β**(1–42)-associated free radical oxidative stress hypothesis to explain oxidative stress under which the MCI and AD brain exist and the loss of synapses in both disorders. A large number of *in vitro* and *in vivo* studies showed that A**β** causes protein oxidation, lipid peroxidation, reactive oxygen species formation, and cell death in neuronal and synaptosomal systems. Methionine located at residue 35 of A**β**(1–42) is an important contributor to the oxidative stress associated with this neurotoxic peptide. In this paper, we summarize studies involving Met-35 of A**β**(1–42). Understanding the role of the single methionine residue of A**β**(1–42) may help in understanding underlying disease mechanisms in AD and MCI.

## 1. Introduction

Alzheimer disease (AD) is an age-related progressive neurodegenerative disease. This devastating disease is characterized by the presence of senile plaques (SP), neurofibrillary tangles (NFTs), and loss of synapses [1]. Although AD has been known for over 100 years, the exact mechanism(s) of AD progression or pathogenesis largely remain unknown. Mutations of *presenilin-1* (PS-1), *presenilin-2* (PS-2), and *APP* genes cause familial AD (FAD). In addition, other genes, such as *apolipoprotein E* allele 4 (*APOE 4*), *clusterin* (CLU, also called apolipoprotein J (APOJ)), *phosphatidylinositol-binding clathrin assembly protein* (PICALM), *endothelial nitric oxide synthase-3*, and *alpha-2-macroglobulin,* have been suggested as risk factors for AD [2–5]. Studies showed that injection of A*β* cores in rat brain leads to AD-like pathology [6].

## 2. Oxidative Stress in AD and Mild Cognitive Impairment Brain

**Figure 1:**
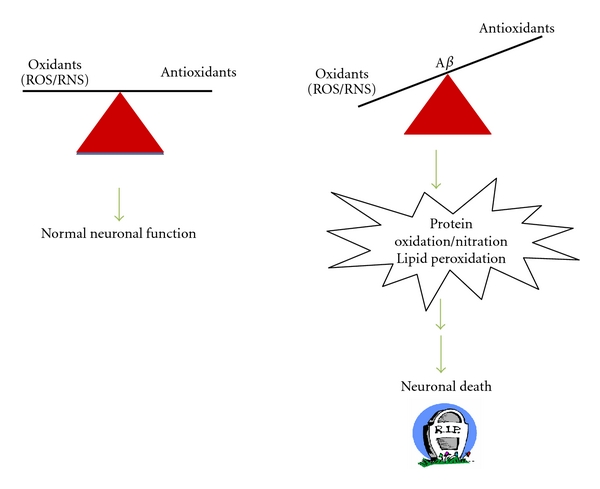
Amyloid beta (A*β*) peptide induces oxidative stress and neuronal loss.

Brain of subjects with AD showed increased markers of oxidative stress [7–10]. A number of hypotheses were proposed to explain AD pathogenesis among which oxidative stress hypothesis is well supported by numerous *in vitro* and *in vivo* studies [11–15]. Oxidative stress is defined as an imbalance between the amount of reactive oxygen species (ROS)/reactive nitrogen species (RNS) and the antioxidant defense system present (Figure 1). Increased levels of ROS/RNS lead to increased damage of biomolecules, such as proteins, lipids, carbohydrates, and nucleic acid. Studies of postmortem tissue showed that AD brain is under extensive oxidative stress, as supported by the elevated levels of biomarkers of protein oxidation, lipid peroxidation, DNA, and RNA oxidation, and advanced glycation end products [10, 16–25]. Further, increased oxidative stress has also been found in MCI [11, 20, 26–30]. The increased markers of oxidative stress in AD and MCI have been shown to be associated with amyloid *β*-peptide (A*β*), a 40 to 42 amino acid residue neurotoxic peptide, that is derived from the proteolytic cleavage of amyloid precursor protein (APP), by the action of beta- and gamma-secretase [31]. A*β*(1–42) has been shown to aggregate more quickly than A*β*(1–40) and is proposed to play a central role in AD pathogenesis. The evidence of involvement of A*β*(1–42) in AD pathogenesis is largely derived from the observation that FAD cases have increased A*β* load and increased oxidative stress. Additional evidence supporting a role of A*β*(1–42) is provided by Down syndrome, characterized by a trisomy of chromosome 21. The gene for APP also is present on chromosome 21, and in Down syndrome individuals, the increase dosage of APP might be responsible for the observation of AD-like pathology in these individuals, if they live long enough. DS brain also has elevated oxidative stress [32].

## 3. Oxidative Stress and A***β***(1–42)

Consistent with the role of A*β* in oxidative stress induction and consequently in AD pathogenesis, our laboratory and others showed that A*β*-induced oxidative stress markers can be alleviated by free radical scavengers such as Vitamin E, melatonin, estradiol, EUK-34, and so forth [33–37]. As discussed further below, our laboratory proposed a model for pathogenesis of AD based on the central role of A*β*(1–42) as a mediator of free-radical-induced oxidative stress in AD brain [23]. In this model, A*β*(1–42) as small oligomers can insert into the lipid bilayer and initiate lipid peroxidation, and, consequently, oxidative damage to proteins and other biomolecules [23]. A sequelae of this model is the influx of Ca^2+^ into the neuron, resulting in loss of intracellular Ca^2+^ homeostasis, mitochondrial dysfunction, and ultimately synapse loss and cell death. 

A*β* exists in different aggregation states, among which oligomeric A*β* is considered as the highly toxic form [38, 39]. Oligomeric A*β* also has been reported to be localized in mitochondria [40, 41], principal foci for ATP generation and a good source of free radicals. *In vitro* studies showed that incubation of 9–11-day-old primary hippocampal neuronal cultures with A*β*(1–42) leads to increased oxidative stress markers and neurotoxicity [14, 42]. Consistent with oxidative stress associated with this neurotoxic peptide, the addition of vitamin E, a chain-breaking antioxidant, significantly modulates the oxidative stress and neurotoxic effects of A*β*(1–42) [33, 42, 43], suggesting that the A*β*(1–42)-mediated toxicity is mediated via its ability to lead to induce oxidative stress. Others showed that depletion of vitamin E increases A*β* accumulation by decreasing clearance from brain and blood in a mouse model of AD [44], and we recently showed that the major efflux protein for A*β*(1–42) from brain to blood, LRP-1, was oxidatively modified in AD brain [25]. Studies conducted in our laboratory using A*β*(1–42), A*β*(1–40), A*β*(17–42), and A*β*(25–35) showed that Met-35 of these peptides is critical for A*β*-induced toxicity and oxidative stress [14, 33, 45, 46]. Many researchers have used A*β*(25–35), which mimics several of the oxidative and neurotoxic properties of full-length A*β*(1–42). The shorter peptide is reported to be more toxic than the regular A*β*(1–42) which could be due to the presence of the methionine residue at the peptide C-terminus. Several studies were conducted to explore the role of C-terminal Met in A*β*(25–35). Further, when the Met C-terminal carboxylic acid functionality of A*β*(25–35) was substituted by an amide (-CONH_2_), the peptide no longer induced any oxidative stress or neurotoxicity, suggesting that the initial attack on the sulfur is important for A*β*-induced oxidative stress and neurotoxicity. In addition, truncation of A*β*(25–35) to A*β*(25–34), diminished oxidative stress and neurotoxicity associated with A*β*(25–35) peptide [47]. However, is should be emphasized that (a) A*β*(25–35) is not found in AD brain, and (b) because the Met is located at the C-terminus in this shorter peptide, while Met is located in a intrachain position of AD-relevant A*β*(1–42), the mechanisms of Met-centered radical cation formation (see below) in the two peptides are completely different. Hence, we recommend that A*β*(25–35) not be used for studies designed to gain insights into mechanisms for A*β*(1–42). Although this review concentrates on Met-35 of A*β*(1–42), other sources of ROS and RNS likely contribute to the oxidative stress observed in AD brain. For example, mitochondrial dysfunction, redox metal reactivity, and so forth, could play an important role in oxidative stress in AD.

## 4. Role of Methionine-35 of A***β***(1–42) in Oxidative Stress

**Figure 2:**
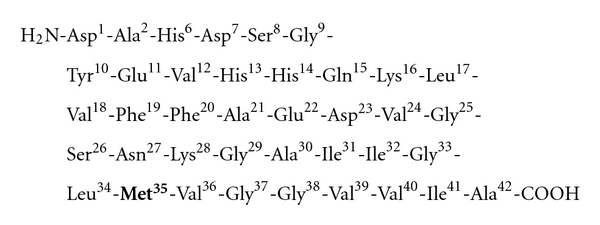
The sequence of amyloid beta (A*β*) peptide. Methione is present in the 35th position of A*β*(1–42).

Our laboratory was the first to propose that A*β*(1–42) mediates its toxicity via the single methionine residue of the peptide [46]. As shown in Figure 2, A*β* has Met present at the 35th residue in the peptide. Met is an easily oxidizable amino acid and can undergo 2-electron oxidation to form methionine sulfoxide [48, 49]. Oxidation of Met has been shown to occur in a wide variety of proteins and has been shown to decrease or hinder biological activity [50, 51]. The oxidation of Met to the sulfoxide could serve two purposes biologically: first, as regulatory function, and secondly, in cellular defense [52]. The notion that Met oxidation to the sufoxide possibly serves an antioxidant role comes from the fact that cell has an enzyme, methionine sulfoxide reductase (MSR), that catalyzes the conversion of Met-sulfoxide (MetSOx) to Met [53–55]. In AD, MSR activity is diminished compared to control brain [56]. Further, the MetSOx can be further oxidized to form a corresponding sulfone, but this process requires a stronger oxidant; hence, this conversion is not easily accomplished *in vivo* and is not an easily reversible process.

The mechanism of free radical oxidation of sulfur in Met-containing A*β* peptides has been extensively studied. In AD brain, a significant fraction of SP-resident A*β* peptide has Met in the form of MetSOx [57]. As noted, previous investigations showed that MSR activity is reduced in AD brain, which may lead to increase oxidation of Met [56]. In addition to 2-electron oxidation, the sulfur atom of Met of A*β*(1–42) can undergo a one-electron oxidation leading to the formation of a sulfuranyl radical cation (S^+·^) or hydroxysulfuramyl radical cation [58]. Further, it appears that H-atom abstraction, likely from the adjacent methyl group on Met, could lead to the formation of an *α*(alkylthio)alkyl radical of methionine, which can react with paramagnetic oxygen to form a highly reactive peroxyl radical or other species such as formaldehyde and sulfhydryl [58];

### 4.1. In Vitro Evidences

To test the role of Met in A*β*-mediated toxicity, the sulfur atom of methionine in A*β*(1–42) was substituted by a methylene moiety (A*β*(1–42) M35NLE) that has the same side chain length and hydrophobicity as Met [14]. The addition of A*β*(1–42M35NLE) to 9–11-day-old primary hippocampal cultures caused no increase in the markers of oxidative stress and neurotoxicity [14]. Further, electron paramagnetic resonance (EPR) spin trapping analyses showed that A*β*(1–42M35NLE) did not lead to trapped free radicals, in marked contrast to native A*β*(1–42), suggesting that that the sulfur atom of Met is key to the A*β*(1–42)-mediated free radical formation. Three laboratories have confirmed our results that substitution of norleucine for Met in A*β*(1–42) significantly modulated, if not eliminated, oxidative stress and neurotoxicity [59–61]. In contrast, another group reported that while the 33–35 region of A*β*(1–42) was critical for the aggregation and neurotoxic properties of this peptide, substitution of Met by norleucine (NL) was still toxic [47]. This different result from our and others' studies could be due to different experimental conditions used in these two studies. For example, in our study we have used aged neurons, that is, 9–11 days in culture (by this time transporters and other key cellular components are fully expressed); in contrast, others [47] used 2-day-old neuronal cultures that still lack the complete expression of the cellular proteome.

### 4.2. In Vivo Evidences

Most of the studies performed to better understand the role of Met of A*β*(1–42) in oxidative stress and neurotoxicity involved *in vitro* studies. However, recently our laboratory performed the first *in vivo* study of a mammalian system to address this issue [62]. The PDAPP mouse is a transgenic mouse in which the human APP gene having mutations corresponding to the Swedish and Indiana familial forms of AD are expressed (APP_Sw, In_) behind a platelet-derived growth factor (PDGF) *β*-chain promoter, resulting in A*β* accumulation, plaque formation, and memory deficits [62]. In our study, a third mutation in APP was introduced, namely, M631, corresponding to Met-35 of A*β*(1–42), was substituted by leucine (M35L) [62]. Leucine was chosen since it is a naturally occurring amino acid with an extant codon, unlike norleucine, which does not have a codon in the cell. Moreover, Leu, like NLE, also exhibits similar length and hydrophobicity as Met. The PDAPPM631L mice showed similar levels of A*β*(1–42) at 9 months of age, the age at which the biochemical and pathological aspects of this study were conducted, as the PDAPP_Sw, In_ mice. However, in contrast to the brain of PDAPP_Sw, In_ human double mutant Tg mice, which contained A*β*-rich plaques, the PDAPPM631L mouse brain presented punctate deposits of A*β*(1–42), suggesting that in addition to affecting oxidative stress, the single Met residue of A*β*(1–42) also is important in plaque formation. Evidence of microglial activation was found in the PDAPPM631L mice. Importantly, the brain of 9-month-old PDAPPM631L mice showed no elevated oxidative stress compared to PDAPP_Sw, In_ mice and compared to WT littermate mice, demonstrating *in vivo* in mammalian brain that the Met-35 residue of A*β*(1–42) is essential for oxidative stress associated with this peptide. Surprisingly, this substitution did not rescue the learning and memory deficits assessed by the Morris water maze at 6 months of age of the PDAPP_Sw, In_ mice. However, to determine if at a later age the deficits in learning and memory observed in the PDAPP_Sw, In_ mice would be modulated in the PDAPPM631L mouse, a longitudinal study would be required. Indeed, as noted above, while indices of oxidative stress and pathology in brain were determined at 9 months of age, behavior necessarily had to be determined at an earlier age, and in our study mice were tested for behavior at 6 months of age. So, it is possible that we missed behavioral changes that would have been manifested later. This notion is consistent with studies of mice with other APP mutations in which behavioral alterations observed later in life (13 months) were not manifested earlier in life [63]. Also, that microglial activation occurred in brain of PDAPPM631L mice may have contributed to the lack of rescue of behavior compared to the PDAPP_Sw, In_ mice. Finally, A*β*(1–42) is but one neurotoxic peptide produced from APP proteolytic processing: other, non-A*β* peptides are produced, that is, sAPP*α*, Jcasp, and C31, each of which has been implicated in neurotoxicity or other components of the AD phenotype [64–67]. Hence, while the oxidative stress in brain of PDAPP_Sw, In_ mice is clearly related to A*β*(1–42), the behavioral aspects inherent in this mouse and the PDAPPM631L mouse conceivably involve one or more of these other APP proteolytic processing products. More research on this topic is necessary. Our laboratory is currently employing proteomics to explore the proteome to better understand the role of Met-35 of A*β*(1–42) in oxidative stress in brain *in vivo*.

The earliest study demonstrating *in vivo* the importance of methionine in A*β*(1–42)-associated oxidative stress involved transgenic *Caenorhabditis elegans* (*C. elegans*). *C. elegans* were genetically modified using an unc-54 promoter (a muscle promoter) to express human A*β*(1–42) which led to the deposition of A*β*(1–42) in the muscle wall of the worm. Increased protein oxidation was found correlated with the phenotypic expression (e.g., paralysis) of the worm [14, 38]. Our result was recently confirmed by others [68]. If in the *C. elegans* that express human A*β*(1–42), Met-35 were substituted by cysteine, no oxidative stress was found, but the deposition of modified A*β*(1–42) was not altered [14]. One caveat to this study is that it is possible that the ability of Cys residues to form disulfide linkages contributed to some degree to the lack of oxidative stress in *C. elegans* that expressed human A*β*(1–42)M35C.

## 5. Methionine and A***β***(1–42) Oligomer

A number of studies also suggest that aggregation of A*β* peptides is essential for determining the toxicity of oxidative stress and neurotoxicity [69, 70]. *In vitro* studies that compared the toxic effect of A*β*(1–42)M35SOx and native A*β*(1–42) showed that the when Met-35 is oxidized to the sulfoxide in A*β*(1–42), the resulting peptide has decreased toxicity assessed 24 h after incubation [45]. A report suggests that oxidation of Met-35 to the sulfoxide makes this A*β* less neurotoxic by altering production of toxic A*β* oligomers that are reported to be the toxic species A*β* [71, 72]. However, others showed that at long incubation times (96 h), A*β*(1–42)M35SOx is still toxic [73]. Likely, mechanisms involved are different than those in which shorter incubation periods (24 h) showed no toxicity of A*β*(1–42)MSOx and may involve aggregated extracellular peptide activating RAGE receptors or other processes.

## 6. Mechanism of Methionine-35 of A***β***(1–42) Toxicity

**Figure 3:**
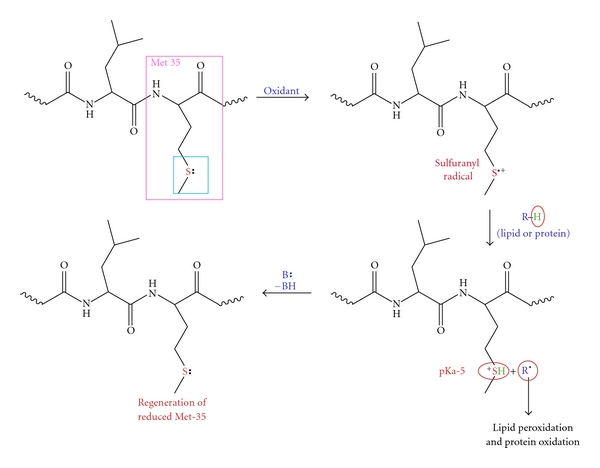
Methionine-35 residues in A*β* can undergo one electron oxidation to form a sulfuranyl radical, which can then initiate the process of lipid peroxidation. For details of this proposed mechanism, please refer to the text.

The proposed A*β*-induced oxidative stress and neurotoxicity is based on the one-electron oxidation of Met-35 to form the sulfuranyl radical cation (Figure 3). The nature of the oxidant is not known for certain, but two candidates are possible among others. Molecular oxygen (zero dipole moment; therefore, soluble in lipid bilayers), would form superoxide radical anion as it oxidized the S atom in Met to the sulfuranyl radical. Superoxide, in turn, could reduce Cu^2+^ to Cu^+^ that is able to catalyze Fenton chemistry to produce reactive and damaging hydroxyl free radicals. Moreover, there is a report that A*β*(1–42) has Cu/Zn SOD-like properties [74], which, if sustained by further studies, would imply that there must be a nearby source of superoxide radicals needed to be scavenged to prevent further damage. The second possible oxidant to convert Met in A*β*(1–42) to a sulfuranyl free radical is Cu^2+^. Copper is shown to bind to A*β* with a high affinity via His present at 6, 13, 14, of A*β*(1–42) [75]. In amyloid plaques, high levels of copper were reported and have been shown to form complex with A*β* [76]. *In vitro* A*β*(1–42) and Cu(II) interaction promotes the reduction of peptide-bound Cu^2+^ to Cu^+^ and form H_2_O_2_·Cu^+^, in turn, can react with the H_2_O_2_ to form hydroxyl free radicals [77, 78]. Moreover, it has been shown that when methionine was added exogenously to a fragment of A*β* that lacks Met (A*β*(1–28)), an increased rate of Cu(II) reduction occurred, supporting the importance of methionine in A*β*-induced toxicity [77, 79]. *In vitro* studies have shown that chelation of copper by a suitable ligand inhibited the formation of ROS. An effective chelator clioquinol (CQ, 5-chloro-7-iodoquinolin-8-ol), hydroxyquinoline antibiotic was found to be an effective high-affinity chelator in blocking the formation of hydrogen peroxide by A*β* [75, 80] and resulted in 85–90% reduction of secreted A*β*-(1–40) and A*β*-(1–42) in Chinese hamster ovary cells overexpressing amyloid precursor protein compared with untreated controls [81]. Further, oral administration of the clioquinol in Tg2576 mice resulted in a reduction of cortical deposition of amyloid (49%) compared to untreated mice [82]. CQ has nanomolar affinity for Cu^2+^ [83], which is sufficient to facilitate dissociation of this metal ion from the low-affinity metal binding sites of A*β*, thereby increasing levels of copper for biological function in the brain of treated animals. Further, a small Phase 2 clinical trial of CQ in moderately severe AD patients for 36 weeks slowed the rate of cognitive decline, and reduced the plasma A*β*42 levels as compared to the placebo controls [84]. However, the role of Cu^2+^ remains unclear. 

Since A*β* is generated by the proteolytic cleavage of APP, which is a transmembrane protein, and A*β* has a hydrophobic C-terminus, we proposed that A*β* peptide, as a small oligomer, insert itself into the lipid bilayer in an *α*-helical conformation. This secondary structure of lipid-soluble A*β*(1–42) is consistent with the secondary structure adopted by the transmembrane segments of most bilayer-soluble proteins. NMR studies conducted on A*β*(1–40) demonstrated that Met residue of A*β* in a micellar environment exists in an *α*-helical conformation [49]. As with all proteins with an alpha-helix conformation, the helix portion of the protein follows the i + 4 rule, that is, every fourth amino acid interacts. Based on the peptide sequence of A*β*(1–42), Met-35 S-atom would interact with carbonyl oxygen of Ile-31 [85]. In this interaction, oxygen being more electronegative than sulfur will pull the electron density towards it making the S-atom in Met-35 more vulnerable to one-electron oxidation, consequently leading to the formation of the methionine-resident sulfuranyl free radical (MetS^+·^; Figure 3) [45, 58, 85]. Since this radical is generated in the lipid bilayer, which is rich in unsaturated fatty acid acyl chains, abstraction of allylic H atoms on these acyl chains initiates the process of lipid peroxidation and resulting in the formation of a carbon-centered lipid radical (L^·^) [86]. The carbon-centered radical can readily react with nonpolar and paramagnetic molecular oxygen to form a lipid peroxyl radical (LOO^·^). Allylic hydrogen atom abstraction from a neighboring lipid acyl chain results in the formation of a lipid hydroperoxide (LOOH) and another carbon-centered lipid radical (L^·^), thus propagating the free radical chain reaction. This reaction will continue unabated as long as there are unsaturated sites with which the lipid acyl chain-resident L^·^ can react. Note that the reaction scheme in Figure 3 has the effect of amplifying the damage caused by radical formation on A*β*(1–42). Moreover, the lipid hydroperoxide formed in this reaction scheme can be decomposed to reactive lipid peroxidation products such as 4-hydroxy-2-nonenal (HNE), 2-propen-1-al (acrolein), and isoprostanes, each of which is found in AD and MCI brain [87]. However, if a chain-breaking antioxidant like vitamin E is present, the chain reaction will be halted and reactive and damaging lipid peroxidation products will no longer be made. This is likely the mode by which vitamin E is able to block ROS formation and neurotoxicity in neuronal systems [33].

Referring to Figure 3, when the allylic H-atom is abstracted by the sulfuranyl free radical, an acid of pKa-5 is formed. This acid can react with any base, for example, water, to lose the H^+^ to form reduced Met again. That is, the whole reaction is catalytic. This model of A*β*(1–42)-induced lipid peroxidation and subsequent oxidative damage to proteins is consistent with the results of theoretical and experimental studies which showed that the *α*-helical secondary structure of the peptide provides stabilization of the sulfuranyl radical formed by a one-electron oxidation of methionine. Further, in vitro studies showed that substitution of Ile-31, that interacts with the sulfur atom of Met-35 by the *i* + 4 rule of a-helices, by proline, an alpha-helix breaker, abrogates the oxidative stress and neurotoxicity associated with A*β*(1–42) [85]. That is, the secondary structure of A*β*(1–42) contributes to reactivity of the neurotoxic peptide. 

That lipid peroxidation requires the radical source to be within a van der Waals distance from the allylic H-atoms with which they react, that lipid peroxidation is well documented in AD and MCI [11, 88], and that A*β*(1–42) directly leads to lipid peroxidation [24], indicates that the radical source to initiate the free radical must be soluble in the bilayer, since free radicals are too reactive to be formed outside the bilayer and diffuse into the bilayer to abstract an allylic H-atom. That is, oligomeric A*β*(1–42) must be soluble within the bilayer to initiate lipid peroxidation. Another sequelae of lipid soluble A*β*(1–42) that adopts an *α*-helix secondary structure conceivably may be related to the greater neurotoxicity of A*β*(1–42) compared to A*β*(1–40), namely, all *α*-helices have an intrinsic dipole moment, which has the effect of stabilizing a radical intermediate, that is, sulfuranyl free radical. Since A*β*(1–42) has two additional amino acids in length than A*β*(1–40), the dipole moment of the former is larger than the latter, and this increased dipole moment conceivable could stabilize the sulfuranyl free radical on Met-35 longer, thereby providing greater opportunity for allylic H-atom abstraction to occur, that is, for lipid peroxidation to be initiated.

Another study showed that substitution of Met35 to Val leads to enhanced toxicity and cellular binding of amyloid peptide [89]. In this study, the authors suggested that substitution of M35 by Val might lead to a change in the conformation of A*β*, that is, change the structure from alpha-helix to a mixture of alpha-helical and beta-sheet conformations, and thereby increasing the binding of Cu^+2^ to this peptide and making it more toxic [89]. Studies from our laboratory conducted by substituting His 6, 13, 14 in A*β*(1–42) by Tyr, which binds Cu^2+^ at least 100-fold less well than does His, showed that this peptide still retains its ability to induce oxidative stress and neurotoxicity emphasizing the importance of Met-35 in the A*β*-induced toxicity and oxidative stress [42, 45]. Also, theoretical studies suggested that Met-mediated reduction of Cu^2+^ found on His residues 6, 13, 14 of A*β*(1–42) would be facilitated by the aromatic pi orbitals of Phe20 [90]. To test this notion, our laboratory substituted aromatic Phe by aliphatic alanine (Ala) and showed that while there was a slight reduction in the oxidative stress and neurotoxicity associated with A*β*(1–42), basically this peptide had quite similar properties as native A*β*(1–42) [42]. 

A computational study from Brunelle et al. reported that the sulfuranyl free radical on Met of one molecule of A*β*(1–42) could abstract an H-atom from the *α*-carbon of Gly-33 of an adjacent peptide in a *β*-sheet conformation. The His-mechanism suggests that the H-atom abstraction would lead to generation of C-centered free radicals which should be able to interact with molecular oxygen leading to the formation of a peroxyl radical and consequently to the protein and other biomolecules damage. Basically this mechanism suggests that no redox metals are necessary to trigger the oxidative stress process [91]. To test this idea, we substituted Gly-33 in A*β*(1–42) by Val (A*β*(1–42G33V)), to prevent the H-atom transfer to Met-35 and observed that this substitution leads to reduced levels of oxidative stress markers and does not cause cell death [92].

## 7. Methionine-35 of A***β***(1–42) Localization in Membrane is Key to Oxidative Stress

Noting that lipid peroxidation by A*β*(1–42) oligomers is critical in our model of A*β*-induced oxidative stress and neurotoxicity, we tested the soundness of this hypothesis. By substitution of negatively charged aspartic acid for glycine residue 37, which is predicted to remove the Met35 residue out of the hydrophobic lipid bilayer, we hypothesized that the sulfuranyl free radical would no longer be resident in the bilayer, and that oxidative stress and neurotoxicity in cells would be prevented [85]. This study supported our hypothesis, and is a direct test of our model (Figure 3), in which lipid solubility of A*β*(1–42) as small oligomers is essential for subsequent oxidative stress and neurotoxicity [7, 87, 93, 94]. Therefore, providing evidence that lipid peroxidation is the initial event in the A*β*-induced toxicity, as demonstrated by us and others [7, 93, 94], would be a major advance in understanding potential roles of this neurotoxic peptide.

In contrast to the previous studies on M35Nle and M35Val substitutions studies discussed above, a recent study suggested that while M35 is important for A*β* assembly, this residue was not important for A*β*-induced toxicity [95]. However, there are a number of differences in the methodologies of this study and those normally employed, one of which is use of a thin film of peptide onto which buffer is added. Oligomerization and deposition of A*β*(1–42) show some dependence on Met-35 of this toxic peptide [62]. Consequently, in addition to the role of Met-35 described in the review, differences in aggregation, location (i.e., membrane bilayer), and role of H-transfer from Gly-33 could play roles in the oxidative stress observed in AD brain. Taken together, most laboratories that have examined the issue have provided evidence for the involvement of A*β*(1–42) in AD oxidative stress (reviewed in [11, 23, 88] and indicated that Met-35-associated formation of free radicals is critical to A*β*(1–42)-induced oxidative stress and neurotoxicity [46, 59–61]. Consistent with this notion, we suggest that either augmenting brain-accessible exogenous antioxidants or upregulating endogenous antioxidants may be promising therapeutic strategies to prevent or delay this devastating disorder. More research is needed to delineate the exact mechanism of A*β*-induced toxicity, and our laboratory is using redox proteomics approaches to identify oxidatively modified proteins and altered pathways involved in brain as a result of A*β*(1–42)-associated, Met-35-mediated oxidative stress that may play a role in pathogenesis and progression of AD.
